# Invisible Scars or Open Wounds? The Role of Mid-career Income for the Gender Pension Gap in Sweden

**DOI:** 10.3389/fsoc.2019.00084

**Published:** 2019-12-20

**Authors:** Stefanie König, Boo E. A. Johansson, Kristian Bolin

**Affiliations:** ^1^Department of Psychology, University of Gothenburg, Gothenburg, Sweden; ^2^Centre for Ageing and Health, University of Gothenburg, Gothenburg, Sweden; ^3^Department of Economics, University of Gothenburg, Gothenburg, Sweden

**Keywords:** pension, gender, financial worries, life course, retirement

## Abstract

This study investigates the importance of mid-career income for the gender pension gap and psychological scarring effects of low income earlier in life. More specifically we analyse whether women's typically less stable mid-life careers also affect outcomes in late careers and in retirement. Swedish income register data from 1990, 2009, and 2015 was linked to the “HEalth, Ageing, and Retirement Transitions in Sweden” survey. The gender pension gap of 966 retirees and worries about pension income of 2,723 older workers between the age of 60 and 66 years were investigated. Blinder-Oaxaca decompositions were applied to analyse the gender pension gap and linear regressions were used for the analysis of financial worries. Results show that gender differences in mid-career income play a stronger role for the gender pension gap than late career income. Mid-career income is furthermore related to higher worries about pension income and accounts for observed gender differences. Our findings demonstrate that gender gaps in mid-career income can be regarded as an open wound with visible negative effects in older ages. The reformed pension system in Sweden may potentially contribute to an even greater gender gap in pensions.

## Introduction

Life course research frequently finds evidence on scarring effects from early career experiences and exposures for later life outcomes (Lucas et al., 2004; Brandt and Hank, 2014; Hetschko et al., 2014; Strandh et al., 2015; Heisig and Radl, 2017). Previous research often investigated unemployment. This study will focus on gender gaps in mid-career income and how they may contribute to gender gaps in pensions as well as gender differences in the subjective expectations of financial difficulties after retirement. Life-time earnings deserve more attention in today's context of aging societies and reformed pension systems. As in the case of Sweden, state pension benefits are often more closely linked to life-time earnings in an effort to reduce the financial pressure on pension systems.

Mid-career earnings often differ between men and women due to women's higher responsibility for the household and childrearing. A selection of women into female dominated occupations and more flexible jobs including part-time work can be observed, in particular in midlife (Hakim, 2016). Our study aims to investigate how these gender differences in midlife are carried forward, throughout the life-course, with a risk to negatively affect economy and well-being of women later in life. Do these midlife gender gaps in fact contribute to scars in late life pension income? Also, do they leave a feeling of financial insecurity and stress in late life and can therefore be seen as an open wound?

The gender earning gap is well-established and often explained by gender differences in occupations, part-time jobs, and resulting career opportunities (Hakim, 2016; Magnusson and Nermo, 2017). It also translates into the gender gap in pension benefits due to the earning-related component in most pension systems (Haitz, 2015). However, more detailed up-to-date research is highly needed since only the most recent retirement cohorts show more comparable careers of men and women. The latest birth cohorts are also affected by the changes in the pension systems which were implemented throughout the late 1990s (Ebbinghaus, 2011; Hofäcker et al., 2016). To make pension systems more financially sustainable, pension benefits were more strongly linked to the market, although individualized to different extents throughout Europe. Thereby, the risk is shifted toward the individual (Blossfeld et al., 2011), leaving certain labor market groups with low pension income. It can be argued that these groups will either need to work longer to accumulate higher pension benefits or will they be faced with more financial hardship after retirement compared to previous cohorts. Among these disadvantage groups are lower educated workers, women, and migrants (Guardiancich, 2012; Peeters and Wouter, 2015; Hofäcker et al., 2016; Neels et al., 2018).

Researching retirement in today's context includes cohorts born from 1949 to 1955 who started working in the 70s. At that time, female careers were much more distinct from men's careers due to a higher prevalence of housewives and final labor market exits of women after family formation. Acknowledging this, it is useful to investigate a country where women had comparatively long careers in this cohort. Sweden has a long history and emphasis on gender equality in labor market issues and was found to show comparatively long careers among women (Lyberaki et al., 2011). Given this background, it is surprising that Sweden in fact demonstrates substantial gender differences in old-age poverty rates in Europe, only exceeded by Slovenia and Bulgaria in 2012 (Haitz, 2015). In 2015, 42.5% of women over 65 years were below the at-risk poverty threshold of having <70% of the median income. Within Europe, this level is only exceeded by Lithuania, Bulgaria, Latvia, and Estonia. Only 23.5% of men in Sweden were below this level. This gender differences ranks also very high across European countries with only Estonia exceeding this gap. Compared to the gender gap of a younger age group between 25 and 54 years (17.1% of men and 17.6% of women are below the threshold), the increase for the age group over 65 years is again among the highest for Sweden. This means that the gender income gap seems to be more strongly affected by the transition into retirement in the Swedish context, compared to most other European countries (Eurostat, 2018). Acknowledging that the 70% threshold is a rather generous criterion and that the pattern for actual poverty rates differs, the dynamics of gender gaps in income become obvious in this example.

The present study makes use of the HEalth, Ageing, and Retirement Transitions in Sweden (HEARTS) survey which covers retirement transitions of 60–66 years olds in a population-based sample. Additionally, register data was linked to the survey, supplying a variety of detailed information on pension benefits and career histories since the year 1990.

## The Swedish Pension System and Preconditions for Pension Benefits

Reforms of the pension system in the late 1990s and the early 2000s changed the preconditions for retirement transitions and income. The old pension system was a defined benefit system and benefits were calculated on the best 15 out of 30 years of contribution (König and Lindquist, 2016). Since highest earning usually occur by the end of the work life, mid-career earnings were irrelevant for the state pension benefits in most cases during the time of the old pension system.

Due to financial pressure on the existing pension system, reforms were introduced in the late 1990s and early 2000s to prolong working lives. The system changed from the principle of “defined benefits” to “defined contributions.” Contributions from the entire working career counted for later pension income instead of the best 15 years, as it was in the previous system. This highlights the importance of life-time earnings and particularly of early career earnings. These changes in the calculation of the earning-related part of the state pension, called income pension, is therefore highly relevant for our research question.

Furthermore, to disburden state pension expenditures, emphasis was placed on private pensions (Ebbinghaus, 2006). Additionally, a shift can be observed in the rising importance of occupational pension while the replacement rates for state pensions decreased from 70% in 1990 to 54% in 2005 (König et al., 2016). For total pension income, state pension benefits contribute with 61% for average earners and even less for high income workers (König et al., 2016). Low mid-career earnings are likely to result into lower occupational pensions, possibly lower private pensions due to fewer saving opportunities and—mainly in the case of the reformed current pension system—into lower state pensions.

Hence, to examine whether the changes in the state pension may play a role for the gender gap, the first hypothesis is on the decomposition of the total pension income.

*Hypothesis 1: Gender differences in state pension benefits play a significant role accounting for the total gender pension gap*.

The gender gap in pension income is well-researched and often related to women's lower lifetime earnings, career interruptions, atypical employment, and part-time periods (Pienta, 1999; Yabiku, 2000; Bardasi and Jenkins, 2002; Evandrou and Glaser, 2003, 2004; Raymo et al., 2010; Hinrichs, 2012; Finch, 2013). Indeed, career histories were found to have a higher explanatory power for women's pension income compared to men's (Möhring, 2015) and were suggested to be related to prolonged working lives for women, potentially for economic reasons (König, 2017; Stafford et al., 2018).

For Sweden, it is argued that periods of childcare or unemployment up to 3 years are well-protected by the particularities of the pension system (Natali and Stamati, 2013) because entitlements are linked to state benefits throughout life (Anxo et al., 2012). However, substantial gender differences in poverty risks among the older population and high gender pension gaps (Haitz, 2015) are still prevalent. One factor may be the comparatively high penalty in pension benefits for part-time work as suggested by a report on “Part-Time Work in the Nordic Region” (Nordic Council of Ministers, 2014). While some women stay in part-time employment throughout their career, a large proportion has temporary part-time periods during the child-rearing phase (Wahrendorf and Siegrist, 2011). Notably, 38% of the female workforce were employed part-time in 1990 and part-time employment among women reached its peak in 1982 with 47% due to changes in the tax system in Sweden (Sundström and Stafford, 1992). The equivalent proportion for men working part-time is much lower and stays below 10% around this time (OECD, 1995). Hence, our cohort may have been strongly influenced by these changes.

Following the earning-related logic of the pension system, we expect mid-career income to have an effect on pension income. However, it is not clear if gender differences in mid-career income are still a relevant component of the gender pension gap or if it is mainly the accumulated gender differences in late-career income that explains most of the pension gap.

*Hypothesis 2: Mid-career income differences play a significant role in accounting for the gender pension gap beyond the effect of late career income*.

The gender pension gap may not only be an economic disadvantage for women, it may also have negative effects on women's psychological well-being. Worries about pension income can be stressful and might even translate into adjusted retirement behavior. It is suggested that women are in higher need to postpone their retirement for financial reasons (Hofäcker et al., 2019) and they may even be more likely to do so if they worry about their financial security after retirement. To analyse whether (anticipated) pension income has any psychological effects, we therefore investigate worries about the financial situation after retirement, although Swedes seem to report less worries about their retirement income in comparison to other European countries (Hershey et al., 2010). This was partly related to generally low income inequalities in Sweden since a high Gini coefficient was related to higher worries. However, as clarified above, income inequalities between men and women are significant, in particular for pension income. Hence, if income inequalities tend to produce worries about retirement income, there may be significant differences between men and women in Sweden.

Another potentially relevant aspect relates to financial literacy. Financial literacy commonly refers to knowledge of personal finances and the application of it (Huston, 2010). Given the age range of the participants in our study (age 60–66 years), low financial knowledge can be expected to be less common due to the state's efforts of providing annual and updated personal information of expected pension benefits. Nevertheless, low financial literacy may be more common among women (Almenberg and Säve-Söderbergh, 2011), which in turn may be related to more frequent and potentially unnecessary worries.

*Hypothesis 3: Women have higher worries about their pension income than men*.

Whether detecting a significant impact of income in 1990 on the gender gap in pensions or not, women may still be prone to be more worried about their later pension income, potentially due to lower early career income and midlife experiences of precarious work. One could argue that there should be no differences between men and women after controlling for current disposable household income. But, precarious work and financial insecurity in earlier careers may have an effect on worries about future income, beyond current (household) income. If this is the case, mid-career income insecurity can be seen as an open wound instead of an invisible scar.

*Hypothesis 4: Mid-career income is negatively related to worries about pension income beyond the effect of current income*.

*Hypothesis 5: Women do not differ from men with regard to worries about pension income after controlling for mid-career income*.

## Data and Methods

Our hypotheses 1 and 2 on pension income were analyzed with a sample of retirees, while hypotheses 3–5 on expected worries about the financial situation in retirement were analyzed with a sample of older workers who are approaching retirement. We used the “HEalth, Ageing, and Retirement Transitions in Sweden” (HEARTS) survey (Lindwall et al., 2017), which was linked to income register data from Sweden. HEARTS has a population-based sample of 5,913 individuals between the ages of 60 and 66 years in Sweden, recruited through a nationally representative sample from the register Statens personadressregister (SPAR). In this article, the first wave of the study was used which was collected in 2015. From the tax register on health insurance and labor force studies (Swedish: *Longitudinell integrationsdatabas för Sjukförsäkrings- och Arbetsmarknadsstudier*—LISA) data from 1990, 2009, and 2015 was retrieved. The dataset provides detailed information on income sources. For this article, the sum of income from paid employment (gross salary) and self-employment (without losses) from 1990 was used as indicator for mid-life earnings. This can be seen as somewhat problematic since the age at this point varies between 35 and 41 years. However, choosing the same age (e.g., income at age 41) is impaired by different effects due to the economic crisis starting in the early 1990s. In 1990, the unemployment rate was still very low, while only 3 years later it reached an all-time peak. Hence, we decided to use the same year as reference instead of the same age. For pension income, detailed information on the different sources was used. Total pension income consists of occupational pensions (Swedish: *tjänstepension*), private pension insurance (Swedish: *privat pensionsförsäkring*), and different parts of state pension benefits, i.e., income pension (Swedish: *inkomstpension*), premium pension (Swedish: *premiepension*), additional pension from the previous old age pension system for cohorts born between 1938 and 1953 (Swedish: *tilläggspension*), and guaranteed pension (Swedish: *garantipension*). Disability pensions were excluded.

The HEARTS study (Lindwall et al., 2017) contributed with the psychological scarring aspect of the gender pension gap, i.e., worries about future pension income among older workers, who approach retirement age. The dependent variable was a question on expectations for retirement. Participants were asked whether they expect that they will feel more stressed due to worse economy after retirement. This item could be answered on a five-point scale ranging from “completely false” (1) to “completely true” (5). Hence, higher values indicated the fear or worry about an insufficient financial situation after retirement. Gender, a binary variable on the presence of children, highest level of education as a linear measure and the relationship status were included as independent variables. The relationship status differentiated between (1) married or in a partnership, (2) single, never married, (3) divorced/separated, (4) widow/widower.

Different methods were applied in order to answer the overall research questions. For the objective aspect of earlier earnings on pension benefits, a Blinder-Oaxaca decomposition of the gender gap in pension income was conducted. The sample for this analysis consisted of 966 retirees who were defined in the register data as “non-working and without information from employers or income from own company” in the register data, who had no missing values on any of the used variables and had a pension income higher than zero.

First, we address the question: How much of the pension gap can be explained by different types of pensions, i.e., state pension, occupational pension, and private pensions. Thereby, one gains a better understanding of the main differences between men and women in terms of their overall pension income. In a second analysis, we conducted a decomposition into mid-career and late career earnings, and occupation.

Next, we focused on the psychological aspects of gender differences in worries about pension income among workers approaching retirement. Ordinary least square regressions (OLS) were conducted in order to find out whether women are more worried about their retirement finances. The sample consisted of 2,723 older workers who were defined as “gainfully employed/self-employed” in the register data. Stepwise regressions on worries about the financial situation after retirement were conducted to see potential changes of the effect for women. In a first step, socio-demographic and individual factors were included, such as the presence of children, partnership status, and educational level. In a second step, current earnings and disposable household income were added. In the final step, we included income in 1990 to study whether this has an effect that goes beyond current earnings and household income.

## Results

First, our results for the decomposition of the overall pension income in 2015 show the expected gap between men and women which is accounted for by differences in types of pensions. The original results on the logarithmic measure for pension income were transformed back to the total amount in Swedish Crowns (SEK, in hundreds). In 2015, men had on average 295,165 SEK in total pension income, while women had 200,733 SEK (see Tables 1, 2). The gap is due to women's lower occupational pensions and state pension benefits. If women had the same amount of occupational pensions, their overall income would increase by 23.5% which seems to be the most important type of pension that drives the overall pension gap. An adjustment of state pensions (income pension, premium pension and *tilläggspension*) to men's level of benefits would increase women's total pension income by 20.5%. This supports hypothesis 1. When it comes to guaranteed pension, women are more likely to receive this type of pension since they are more likely to have insufficient income pension. Hence, lowering women's income from guaranteed pension to men's levels would furthermore increase the total pension gap. Income differences from private pension do not play a significant role in accounting for the overall pension gap.

**Table 1:** Descriptives for retired men (*n* = 392).

**Variable**	**Mean**	**SD**	**Min**	**Max**	**%**
Log pension (total)	7.710	0.927	2.079	9.574	
Pension (total)	2951.645	1950.188	8	14,381	
Income pension	858.985	485.868	0	1,583	
Occupational pension	1517.908	1714.714	0	12,052	
Tilläggspension	268.946	181.299	0	656	
Premium pension	69.071	45.418	0	238	
Guaranteed pension	9.985	52.131	0	541	
Private pension	226.740	509.837	0	4,991	
Income in 1990	1981.474	913.812	0	5,399	
Income in 2009	3195.941	3127.097	0	34,797	
Managers					11.73
Professionals					17.35
Technicians and associate professionals					21.94
Clerical support workers					5.87
Service and Sales workers					5.61
Agriculture, forestry and fishery workers					0.26
Craft and related trades workers					13.78
Plant and machine operators					9.18
Elementary occupations					4.85
Military and uncategorized					9.44

**Table 2:** Descriptives for retired women (*n* = 574).

**Variable**	**Mean**	**SD**	**Min**	**Max**	**%**
Log pension (total)	7.315	0.959	0.000	9.994	
Pension (total)	2007.326	1518.591	1	21,888	
Income pension	661.221	381.406	0	1,533	
Occupational pension	831.037	1325.788	0	20,411	
Tilläggspension	231.983	153.825	0	560	
Premium pension	57.636	38.990	0	195	
Guaranteed pension	32.160	83.448	0	833	
Private pension	193.298	284.303	0	1,743	
Income in 1990	1139.204	659.481	0	5,359	
Income in 2009	1992.599	1903.363	0	21,134	
Managers					5.40
Professionals					17.77
Technicians and associate professionals					18.99
Clerical support workers					14.98
Service and sales workers					23.87
Agriculture, forestry and fishery workers					0.35
Craft and related trades workers					0.35
Plant and machine operators					3.48
Elementary occupations					5.57
Military and uncategorized					9.23

Explaining the gap by later career characteristics, i.e., income in 2009, women's pension would only increase by 13% if they had the same income as men. This does not even account for half of the existing pension gap. Hence, even if women earned as much as men by the end of their career, the gap between men's and women's pension income would still be rather high due to lower lifetime earnings. With regard to occupational group, women are less likely to be managers (see Table 3), which explains a significant part of the gap (**Table 5**). They are more likely to be in occupations related to clerical work which also slightly contributes to the pension gap. In total, women's income would increase about 6% if they had the same occupations as men.

**Table 3:** Gender distribution across occupational groups for the retired sample (*n* = 966).

	**Men (%)**	**Women (%)**
Managers	59.74	40.26
Professionals	40.00	60.00
Technicians and associate professionals	44.10	55.90
Clerical support workers	21.10	78.90
Service and sales workers	13.84	86.16
Agriculture, forestry and fishery workers	33.33	66.67
Craft and related trades workers	96.43	3.57
Plant and machine operators	64.29	35.71
Elementary occupations	37.25	62.75
Military and uncategorized	41.11	58.89

Adding data on income in 1990 changes the picture drastically. Increasing women's earnings to men's earnings in 1990, women would have a 25.3% higher pension income (see **Table 6**). This explains more than half of the gap and supports hypothesis 2. Income in 1990 (when participants were between 35 and 41 years old) is therefore a good predictor for life-time earnings throughout the working career, and even beyond retirement.

Following these insights, we were interested in the long term subjective effects of low income in earlier careers. In a first model, OLS regressions were used to analyse worries about future pension income, only including individuals still at work (see Table 4). Higher values in the dependent variable indicate higher agreement that the economic situation after retirement is perceived as more stressful. In the first model, education, the presence of children, partnership status, and occupational group were included. The results show that even after controlling for these variables, women worry significantly more about their pension income, which provides evidence for hypothesis 3 (see Model 1, Table 7).

**Table 4:** Descriptives for the working sample (*n* = 2,723).

**Variable**	**Mean**	**Std. dev**.	**Min**	**Max**	**%**
Women					55.2
**Educational level**					
<9 years					1.1
Elementary education (Grundskola)					9.4
Occupational education (Yrkesutbildning)					21.5
Gymnasium					11.2
Post-gymnasium education (Eftergymnasial utbildning)					9.6
University level without exam (Högskoleutbildning/ej examen)					8.7
University degree (Högskole/universitetsexamen)					38.5
No Children					8.5
**Partnership status**					
Married/partner					74.0
No partner					8.0
Divorced					14.9
Widow/er					3.1
**Occupational group**					
Managers					10.6
Professionals					23.2
Technicians and associate professionals					21.8
Clerical support workers					8.7
Service and sales workers					5.0
Agriculture, forestry and fishery workers					1.1
Craft and related trades workers					17.1
Plant and machine operators					8.0
Elementary occupations					2.3
Military and uncategorized					2.2
Household income 2015	6058.951	4365.732	231	6,9284	
Income 2015	3950.865	2474.62	0	3,8965	
Income 1990	1441.938	793.9494	0	6,494	
Worries about financial situation in retirement	2.219611	1.178218	1	5	

**Table 5:** Decomposition of the gender pension gap (logarithmic form) by pension type (*n* = 966).

	**Coef**.	**Robust SE**	***p***	**(95% Conf. interval)**	
**Differential**					
Prediction: men	7.710	0.047	0.000	7.618	7.801
Prediction: women	7.315	0.040	0.000	7.236	7.393
Difference	0.395	0.062	0.000	0.274	0.516
**Explained**					
Occupational pension	0.235	0.047	0.000	0.144	0.327
Income pension	0.151	0.029	0.000	0.093	0.209
Tilläggspension	0.032	0.010	0.002	0.012	0.052
Premium pension	0.019	0.008	0.023	0.003	0.035
Guaranteed pension	−0.020	0.007	0.002	−0.033	−0.007
Private pension	0.013	0.011	0.253	−0.009	0.034
Total	0.429	0.061	0.000	0.310	0.549

**Table 6:** Decomposition of the gender pension gap (logarithmic form) by career characteristics (*n* = 966).

	**Coef**.	**Robust** ** SE**	***p***	**(95% Conf. interval)**	
**Differential**					
Prediction: men	7.710	0.047	0.000	7.618	7.801
Prediction: women	7.315	0.040	0.000	7.236	7.393
Difference	0.395	0.062	0.000	0.274	0.516
**Explained**					
Income in 1990	0.253	0.041	0.000	0.172	0.334
Income in 2009	0.126	0.032	0.000	0.063	0.189
Managers	0.016	0.006	0.012	0.004	0.029
Professionals	0.001	0.003	0.866	−0.007	0.006
Technicians and associate professionals	0.004	0.004	0.315	−0.004	0.030
Clerical support workers	0.014	0.008	0.087	−0.002	0.031
Service and sales workers	0.017	0.012	0.156	−0.006	0.041
Agriculture, forestry and fishery workers	−0.000	0.001	0.799	−0.001	0.001
Craft and related trades workers	0.006	0.011	0.628	−0.017	0.028
Plant and machine operators	0.003	0.004	0.405	−0.005	0.011
Elementary occupations	0.002	0.003	0.625	−0.005	0.008
Military and uncategorized	−0.001	0.006	0.914	−0.012	0.011
Total	0.441	0.053	0.000	0.337	0.545

**Table 7:** OLS regression results on financial worries about future pension income.

	**Model 1**	**Model 2**	**Model 3**
Women	0.206^***^	0.149^***^	0.0205
	(0.0498)	(0.0503)	(0.0547)
Education	0.0441^***^	0.0577^***^	0.0526^***^
	(0.0149)	(0.0149)	(0.0148)
No children	−0.0121	−0.0707	−0.0374
	(0.0910)	(0.0906)	(0.0902)
**In partnership (ref)**			
Single	0.141	0.0814	0.0858
	(0.0944)	(0.0945)	(0.0939)
Divorced	0.330^***^	0.257^***^	0.240^***^
	(0.0633)	(0.0651)	(0.0648)
Widow	0.0586	−0.0115	−0.0195
	(0.128)	(0.128)	(0.127)
**Occupation: manager (ref)**			
Professionals	0.0995	−0.00190	0.0120
	(0.0833)	(0.0841)	(0.0836)
Technicians and associate professionals	0.280^***^	0.133	0.113
	(0.0836)	(0.0859)	(0.0854)
Clerical support workers	0.304^***^	0.145	0.127
	(0.105)	(0.107)	(0.106)
Service and sales workers	0.543^***^	0.351^***^	0.287^**^
	(0.123)	(0.126)	(0.126)
Agriculture, forestry and fishery workers	0.670^***^	0.493^**^	0.499^**^
	(0.222)	(0.222)	(0.221)
Craft and related trades workers	0.552^***^	0.366^***^	0.305^***^
	(0.0951)	(0.0987)	(0.0987)
Plant and machine operators	0.529^***^	0.332^***^	0.267^**^
	(0.110)	(0.113)	(0.113)
Elementary occupations	0.573^***^	0.394^**^	0.310^*^
	(0.167)	(0.167)	(0.167)
Military and uncategorized	0.0982	−0.0603	−0.123
	(0.166)	(0.167)	(0.166)
Household disposable income 2015		−2.83e-05^***^	−2.62e-05^***^
		(5.85e-06)	(5.83e-06)
Income 2015		−3.71e-05^***^	−1.73e-05
		(1.05e-05)	(1.10e-05)
Income 1990			−0.000203^***^
			(3.52e-05)
Constant	1.527^***^	1.967^***^	2.290^***^
	(0.107)	(0.124)	(0.135)
Observations	2,723	2,723	2,723
*R*-squared	0.046	0.063	0.075

*Standard errors in parentheses*.

*** *p < 0.01*,

** *p < 0.05*,

* *p < 0.1*.

In a second model, the current economic situation was included. After controlling for household income and earnings in 2015, the effect for women decreases but remains significant.

In a third model, income in 1990 was added which is significantly related to worries about pension income. This provides evidence for hypothesis 4. Furthermore, in model 3, the effect for women disappears completely as hypothesized (hypothesis 5).

## Conclusion and Discussion

Our overall findings highlight the importance of mid-career income, both for the gender pension gap and in explaining worries about pension income, even after controlling for late career income.

The earning gap between men and women around their late 30s seems to be carried forward throughout the later life course. Even if women “catch up” until the end of their careers, it still cannot compensate for previous earning losses and potentially a feeling of financial precariousness. After controlling for household income and individual income, the negative effect of mid-career income is still highly significant for worries about pension income. The results suggest that women worry more about their financial situation than men, but only before controlling for mid-career earnings. Keeping mid-career earnings constant, women do not differ significantly from men in terms of stressful feelings about future finances. This indicates that potential gender differences in financial literacy do not seem to play a role for income worries. In this sense, gender differences in earlier careers can still be seen as an open wound in later life since it accounts for the variance in worries beyond the effect of late life earnings. Linking those results to the decomposition results, these worries seem to be justified, given the relatively large importance of earlier earnings for total pension income.

Our study provides evidence on the extent of the effect of mid-life earnings on the gender gap in pensions and the financial worries about future pension income. The results have a suggestive meaning for potential effects of the pension reforms in the early 2000s, even though this study does not directly compare pre- and post-retirement gender gaps. In the previous pension system, only the best 15 years of highest earnings were used to calculate pension benefits which offered women a better chance to increase their income to more similar levels than men's by the age around 50. In the reformed pension system of today, benefits are based on the entire career earnings. Hence, differences in starting salaries and earlier careers are now more important and detrimental for the accumulation of pension benefits in the reformed system. Furthermore, the increased relative importance of occupational pensions throughout the life course of the cohort in this study contributes further to the gender gap. Even if women had the same state pensions as men, their overall pension income would still be significantly lower due to the gender differences in occupational pensions.

This study is impaired by the limitations of the relatively young sample. It includes retirees who are younger than 67 years. A recent report on the gender pension gap in Sweden, however, finds that occupational pensions explain more of the gap among younger cohorts, compared to older cohorts. Furthermore, they are also more important for younger retirees compared to older retirees. Hence, the importance of occupational pensions for the total pension gap may be less in a sample with retirees who are 67 years and older. While the results can be expected to be representative for retirees in the age group 60–66 years, they may not be generalizable for the entire population of retirees in Sweden. In our analysis of older workers' expected financial worries, our young sample excluded older workers beyond age 67. Although people in Sweden typically retire before age 67, our results may not be generalizable for those who retire at a later age. Furthermore, there might be a selection bias in our worker sample since those who do not worry about their finances may already be retired. The proportion of people who continue working for financial reasons is relatively low in Sweden (Hofäcker et al., 2019), and we should consequently not expect a strong selection effect. Last, our dependent variable on worries about the financial situation is limited due to the single item measure. Future research should adopt a more differentiated measure of worries and stress to capture this more complex construct.

The political implications of the results go beyond later life and stretch over the entire life course. While there is greater awareness of the effects of occupational choice and part-time work on current income, the consequences for pensions and financial worries in later life are often not considered. Raising awareness in future generations may be a first step to improve financial decision making early in the life-course and thereby decrease the gender pension gap. In Sweden, first awareness-rising campaigns were conducted, providing information on the potential effects of marriage, small children, job changes, and self-employment for later pension benefits (Pensionsmyndigheten, 2017). The Swedish pension system offers an option to decrease the negative effects of these decisions on a couple's level. Pension rights from the premium pension can be transferred from one spouse to the other to compensate for lower pension benefits due to an unequal division of paid and unpaid work. Similarly, private saving arrangements by one partner for the other may help to decrease the gender pension gap and the significantly higher worries about future pension income for divorced individuals.

## Data Availability Statement

The datasets generated for this study will not be made publicly available due to data use restrictions.

## Ethics Statement

The studies involving human participants were reviewed and approved by the ethical approval board of the University of Gothenburg (Dnr: 970-14). The patients/participants provided their written informed consent to participate in this study.

## Author Contributions

This article includes both psychological and economical aspects and benefited from the collaborative effort between SK and BJ from the Department of Psychology and KB from the Department of Economics. SK conducted the analyses and was responsible for the first draft of the article. BJ contributed with input from a psychological perspective and the data preparation. KB contributed with input from an economics perspective. BJ, KB, and SK contributed to the final draft of the article.

### Conflict of Interest

The authors declare that the research was conducted in the absence of any commercial or financial relationships that could be construed as a potential conflict of interest.
